# In Silico Model for Chemical-Induced Chromosomal Damages Elucidates Mode of Action and Irrelevant Positives

**DOI:** 10.3390/genes11101181

**Published:** 2020-10-11

**Authors:** Yurika Fujita, Osamu Morita, Hiroshi Honda

**Affiliations:** 1 R&D Safety Science Research, Kao Corporation, Haga–Gun, Tochigi 321-3497, Japan; morita.osamu@kao.com; 2 Institute for Protein Research, Osaka University, Suita, Osaka 565-0871, Japan

**Keywords:** in silico prediction model, chromosomal aberration, generalized linear model, organic functional groups

## Abstract

In silico tools to predict genotoxicity have become important for high-throughput screening of chemical substances. However, current in silico tools to evaluate chromosomal damage do not discriminate in vitro-specific positives that can be followed by in vivo tests. Herein, we establish an in silico model for chromosomal damages with the following approaches: (1) re-categorizing a previous data set into three groups (positives, negatives, and misleading positives) according to current reports that use weight-of-evidence approaches and expert judgments; (2) utilizing a generalized linear model (Elastic Net) that uses partial structures of chemicals (organic functional groups) as explanatory variables of the statistical model; and (3) interpreting mode of action in terms of chemical structures identified. The accuracy of our model was 85.6%, 80.3%, and 87.9% for positive, negative, and misleading positive predictions, respectively. Selected organic functional groups in the models for positive prediction were reported to induce genotoxicity via various modes of actions (e.g., DNA adduct formation), whereas those for misleading positives were not clearly related to genotoxicity (e.g., low pH, cytotoxicity induction). Therefore, the present model may contribute to high-throughput screening in material design or drug discovery to verify the relevance of estimated positives considering their mechanisms of action.

## 1. Introduction

In silico prediction tools for toxicological evaluations have become increasingly important owing to the demand for high-throughput evaluation in drug discovery and chemical substance design without animal testing. Especially in the cosmetics field, efficient evaluation using in vitro and in silico methods is required to achieve high predictivity of chemical toxicities [1,2], since animal testing is no longer available [3].

Genotoxicity is an important endpoint to predict the carcinogenicity of chemicals [4]. In general, bacterial reverse mutation assays (especially the Ames test) and in vitro mammalian cell tests that were developed to evaluate gene mutations and chromosomal damages are commonly used in a battery evaluation to achieve high sensitivity for carcinogenicity predictions [5]. Although genotoxicity is normally a knockout criterium, in vitro mammalian cell tests sometimes detect in vitro-specific positives, which are misleading or irrelevant positives [5]. Thus, in vivo studies, such as in vivo micronucleus tests, have been used to follow up misleading positives. However, they are low-throughput and have been restricted in terms of animal welfare. Thus, to verify misleading positives, in silico tools that can immediately identify structural alerts in target chemicals are considered promising alternatives to in vivo follow-up studies. Nevertheless, current models for chromosomal damage do not focus on misleading (and irrelevant) positives. Moreover, chemical features related to misleading positives are still unclear. Therefore, models that can predict misleading positives and provide modes of actions are needed for the preparation of adequate follow-up approaches during the early stages of research and development.

Consideration of misleading positives in in vitro mammalian cell tests may also increase the accuracy of in silico tools. Whereas in silico tools for the Ames test show acceptable performance and are used for the genotoxicity evaluation of impurities or by-products [6], in silico tools for the in vitro test for chromosomal damages do not have sufficient predictivity [7]. Morita et al. reported the prediction performance of the current in silico tools for chromosomal damages [7]. In their paper, although MultiCase showed the highest sensitivity among in silico tools, low specificity was reported for both in vitro and in vivo micronucleus test prediction [7], implying a trade-off relationship [8], likely caused by the quality of the training data [2,7,9,10]. Misleading positive chemicals, which are not genotoxic substances, are included in the positive compound list; hence, this noise can affect the performance of in silico tools. Therefore, discrimination between misleading positives and positives in training sets may improve performance.

Morita et al. [11] and Kirkland et al. [12] reviewed several databases and summarized positive, negative, and misleading (and irrelevant) positive chemicals on the basis of weight-of-evidence approaches and expert judgments [11,12]. Furthermore, the Organisation for Economic Co-operation and Development (OECD) test guidelines for in vitro mammalian cell tests were improved in 2014 to avoid misleading test conditions [13,14]. Using this updated guideline, Fujita et al. successfully recategorized misleading positives that were probably caused by cytotoxicity from positives listed in Morita et al. [11,15,16]. However, recent reports that discriminate misleading positives have not been applied to the development of in silico tools for chromosomal damage.

In this study, to construct a useful and precise in silico model that enables the discrimination of positives and misleading positives, we reclassified training data from only two categories (positives and negatives) into three categories (positives, negatives, and misleading positives) according to reliable data sources examined by experts [11,12], which are described above. Subsequently, a generalized linear model (GLM) with L1/L2-regularized logistic regressions, which has been used to identify important molecules and predict toxicity [17,18], and partial structure information (organic functional groups (OFGs)) of each chemical were adopted to identify important structural features of positives and misleading positives. 

## 2. Materials and Methods

### 2.1. Data set of Chemical Substances

#### 2.1.1. Data Acquisition and Classification

In total, 317 chemicals were obtained from recent reports, including Morita et al. [11] and Fujita et al. [15,16,19] (248 chemicals) and Kirkland et al. [12] (69 chemicals) that listed high-quality data (i.e., according to or similar to OECD good laboratory practice [GLP] study) via extensive review. After omitting two duplicated chemicals among the 317 chemicals, 315 chemicals were classified into three categories (positives (108 chemicals), negatives (157 chemicals), and misleading (irrelevant) positives (50 chemicals)) as follows (Table S1 in Supplementary Materials).

1. Positives: (a) chemicals with “positive results in in vitro mammalian cell genotoxicity tests” in Kirkland et al. [12] (25 chemicals) and (b) positive chemicals (70 chemicals) and “chemicals with minimal or some concern” (12 chemicals) in Morita et al [11]. Although o-Dichlorobenzene (CAS No. 95-50-1) had been classified into “missed chemicals with negligible concern” in Morita et al. [11], it was recategorized into positives because positive results were recently reported in both in vivo [20] and in vitro [15] micronucleus tests under the current OECD test guidelines [14]. In total, 108 chemicals were classified as positives.
2. Negatives: (a) chemicals with “negative results in in vitro mammalian cell genotoxicity tests” in Kirkland et al. [12] (27 chemicals) and (b) negative chemicals in Morita et al. [11] (132 chemicals). Since two chemicals existed in both data sets, 157 chemicals were assigned as negatives.
3. Misleading positives: (a) chemicals that “should give negative results in in vitro mammalian cell genotoxicity tests, but have been reported to induce gene mutations in mouse lymphoma cells, chromosomal aberrations, or micronuclei, often at high concentrations or at high levels of cytotoxicity” in Kirkland et al. [12] (17 chemicals), (b) “chemicals with negligible concern” (25 chemicals) in Morita et al. [11], and (c) among chemicals with negative Ames tests in Morita et al. [11], chemicals that were suggested to be misleading positives owing to cytotoxicity [16] and showed negative retest results using in vitro micronucleus test in Fujita et al. [15] (8 chemicals). In total, 50 chemicals were classified as misleading positives. Basically, misleading positive chemicals do not induce genotoxicity in in vivo conditions and induce irreverent positives in in vitro conditions.

#### 2.1.2. Reselection of Chemicals via OFG Extraction

To understand the chemical structure related to positives or misleading positives, OFGs were employed as experimental variables in a GLM. Using QSAR toolbox version 3.4.0.17 [21], OFGs for all evaluated chemicals were exported as a matrix. Names of OFGs were kept as the original names derived from QSAR toolbox in order to search toxicological information in QSAR toolbox later. Since 15 chemicals did not have OFGs (these chemicals showed “No functional group found”), they were eliminated (CAS Nos. 10108-64-2, 15663-27-1, 7784-46-5, 10022-68-1, 7789-12-0, 7803-57-8, 8007-18-9, 7550-35-8, 39430-27-8, 13472-30-5, 7756-94-7, 122852-42-0, 688046-61-9, 13939-25-8, and 7782-63-0). Finally, 300 chemicals (Table S1 in Supplementary Materials; 102 positives, 150 negatives, and 48 misleading positives) were used to develop the prediction model.

### 2.2. Prediction Model Development

A GLM that weighed explanatory variables was employed as a statistical model to identify important OFGs (explanatory variables in this study) related to positives and misleading positives. Glmnet [22] in R packages [23] was used for model development. An odds ratio (OR) was used to analyze the importance of OFGs in predicted results [24]. According to Szumilas et al. [24], OR = 1 indicates that “exposure did not affect odds of outcome”, OR > 1 indicates that “exposure was associated with higher odds of outcome”, and OR < 1 indicates that “exposure was associated with lower odds of outcome.” Two hyper parameters on glmnet were used to derive L1/L2-regularized logistic regressions (elastic net regressions), alpha and lambda, which were optimized in the following scheme. The value of alpha that decides the number of explanatory variables was selected automatically between 0.5 and 1 at intervals of 0.1, which showed the minimum mean squared error (MSE). According to previous knowledge, we confirmed that the number of OFGs as variables was within one-fifth of total chemicals (60 out of 300), to avoid overfitting [25]. The value of lambda was set after the 5-fold validation using cv.glmnet (a function in glmnet).

Imbalanced data can sometimes cause biased predictions (i.e., 100% sensitivity and 0% specificity). In fact, sensitivity using our imbalanced data (102 positives, 150 negatives, and 48 misleading positives) preliminarily showed 5.9% for positives, 100% for negatives, and 0% for misleading positives. To solve this problem, the synthetic minority oversampling technique (SMOTE) in the DMwR [26] in R was adopted. SMOTE can conduct over- and undersampling based on the same chemical categories using the nearest neighbors method. Components of synthesized chemicals by SMOTE are similar to original data sets on the basis of the concept of SMOTE technique [27]. The number of chemicals was set to the maximum number of the three categories (=150). This means that positives and misleading positives were synthesized against negatives. Note that statistical testing was not directly possible for the glmnet method because no standard errors for parameters were calculated directly [28]. Finally, 10-fold internal cross-validations were conducted against the data set after SMOTE treatment using the optimized hyper parameters. Moreover, a model accuracy for the original data sets was also calculated. After OFGs were extracted for each model, we searched toxicological information using the names of the OFGs in the literature. The graphical flow of in silico modeling in this study is shown in Figure S1. 

### 2.3. Performance Evaluation of Models

According to previous studies [29,30], the confusion matrix was evaluated using the parameters below.

Sensitivity (%) = (number of chemicals correctly classified for target class [A])/(number of chemicals in the target class) × 100(1)

Specificity (%) = (number of chemicals correctly classified for non-target class [B])/(number of chemicals in non-target class) × 100(2)

Accuracy (%) = (A + B)/(number of all chemicals) × 100(3)

### 2.4. Visualization of Structural Alerts (OFGs)

To easily understand structural alerts related to positives and misleading positives, we illustrated virtual poly-clastogens using OFGs of the top 20% of OR by referring to the poly-carcinogen illustrated by Ashby et al. [31,32]. In detailed visualization, we combined selected OFGs, and illustrated poly-clastogens for positives and misleading positives using a drawing tool in the OECD QSAR toolbox.

## 3. Results

### 3.1. Prediction Performances of Developed Model

After SMOTE treatment, 150 positive, 144 misleading positive, and 150 negative chemicals were used for model development. In this model using the updated data set, the minimum MSE was observed when alpha was set to 0.5. The prediction performance (mean) in 10-fold internal cross-validation is shown in Table 1. Regarding accuracy, each model showed 85.6% for positives, 80.3% for negatives, and 87.9% for misleading positives (Table 1). Sensitivities of positives, negatives, and misleading positives in this model were 72.6%, 71.0%, and 71.6%, respectively (Table 1). In the analysis that focused on original data sets in cross-validation, accuracies were calculated as 81.6% for positives, 74.1% for negatives, and 87.0% for misleading positives. Among 166 explanatory variables, 60, 52, and 36 variables were selected for positive, negative, and misleading positive predictions, respectively, and each number of explanatory variables was under one-fifth of the chemical number.

### 3.2. OFGs Related to Test Results

Regarding positive predictions, 47 OFGs with positive correlations and 13 OFGs with negative correlations were identified. As OR is “a measure of association between exposure and outcome” [24], and although no standard errors for parameters can be calculated directly in glmnet [28], it can be expected that a higher OR is correlative to a higher contribution to results, as suggested in previous studies [33,34]. The OFGs identified were sorted in descending order of OR. The top 20% of OFGs with positive correlations (Epoxide, Fused unsaturated carbocycles, AlkoxySilane, Sulfonate ester, Fused heterocyclic aromatic, N. Nitroso, Amidine, Isocyanate, and Dianilines), and the main toxicological effects or mechanisms likely related to the positive results, are summarized in Table 2. The suggested and/or reported mechanisms were as follows: (a) structures with Epoxide [35], AlkoxySilane [11], Sulfonate ester [35], and N. Nitroso [35] induced DNA binding, and those with Isocyanate [35] induced DNA acylation; (b) chemicals containing a part of Amidine [36] were DNA minor groove binders; and (c) structural alerts for a part of metabolites of Fused unsaturated carbocycles [21] and Dianilines themselves [21] induced DNA binding. In addition, chemicals with Fused heterocyclic aromatic [35] induced DNA intercalation.

**Figure 1:**
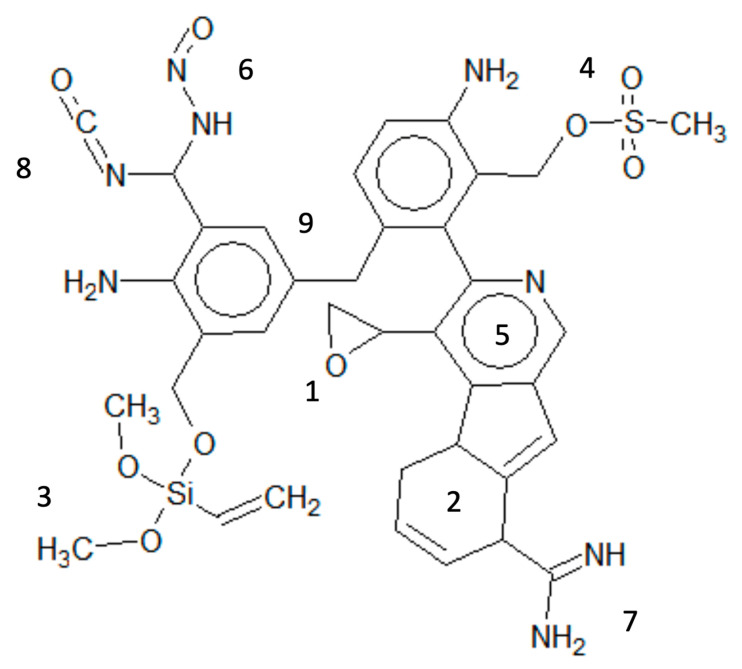
Virtual poly-clastogens derived from OFGs related to positives. Poly-clastogens were described on the basis of Table 2. The names of OFGs are as follows: (1) Epoxide, (2) Fused unsaturated carbocycles, (3) Alkoxysilane, (4) Sulfonate ester, (5) Fused heterocyclic aromatic, (6) N. Nitroso, (7) Amidine, (8) Isocyanate, and (9) Dianilines.

**Figure 2:**
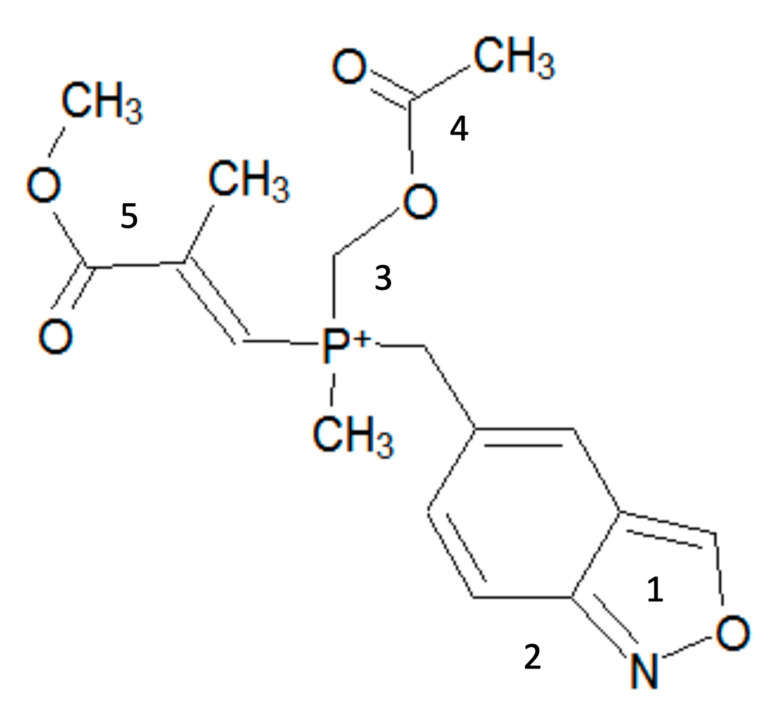
Virtual poly-clastogens derived from OFGs related to misleading positives. Poly-clastogens for in vitro-specific positives are described in Table 3. The names of OFGs are as follows: (1) Oxazole/Izoxazole, (2) Benzthiazolinone/Benzoisothiazolinone, (3) Phosphonium and salt, (4) Acetoxy, and (5) Methacrylate.

In the same manner, 20 OFGs with positive correlations and 16 OFGs with negative correlations were identified for misleading positive predictions. The top 20% of OFGs related to positive correlations (Oxazole/Izoxazole (also generally known as Isoxazole), Benzthiazolinone/Benzoisothiazolinone (also generally known as Benzothiazolinone), Phosphonium, salt, Acetoxy, and Methacrylate) and the main toxicological effects or mechanisms likely related to the misleading positive results are summarized in Table 3. The suggested and/or reported mechanisms were as follows: (a) chemicals containing a portion of Oxazole/Izoxazole [37] or Acetoxy [11] induced anti-tuberculosis activity or low pH, respectively; (b) structures with Benzthiazolinone/Benzoisothiazolinone [38], Phosphonium, salt [39], or Methacrylate [11] induced reactions with the amino groups of lysine residues, cytotoxicity, or in vitro-specific DNA reactivity, and/or cytotoxicity, respectively. The virtual poly-clastogens for positives and misleading positives are illustrated in Figure 1 and Figure 2, respectively. The OR and number of OFGs related to positives were higher than those related to misleading positives. Moreover, whereas OFGs related to positives would generally be involved in various known mechanisms of genotoxicity, OFGs related to misleading positives could be involved in non-genotoxic modes of action.

In the present study, feature selection was conducted via elastic-net regression. The coefficients of the elastic-net model, those of the ridge model that do not perform covariate selection (α = 0), and the correlation coefficients are shown in Table S2 (Supplementary Materials). The elastic-net model did not select OFGs with extremely low correlation coefficients, which could affect model robustness. On the other hand, OFGs that were not selected by the elastic-net despite their high values in ridge regression may be confirmed carefully by expert judgement.

## 4. Discussion

We developed a precise model (accuracy: ≥80.3%) that can predict results of in vitro mammalian cell tests especially with regard to detecting chromosomal damages, including misleading positives, with high sensitivity and specificity using the updated database. Statistical analysis revealed the OFGs and their quantitative importance (OR) that contributed to the induction of positives and misleading positives. The structures identified contained structures that were previously reported in mechanism investigations [11,39], indicating the validity of our approach. In addition, connecting their OFGs to existing knowledge helped us to understand and interpret the mechanisms of action for the induction of positives and misleading positives.

Genotoxicity tests have been developed for hazard identification of chemicals [40]; therefore, sensitivity tends to be more important than specificity [41,42]. The sensitivity of our in silico model to predict positives (72.6%) was close to those of in vitro mammalian cell tests (for genotoxic carcinogens: 82.6%) and existing in silico tools (for positives: 56–91%) [7,43]. In addition, our model showed a more balanced prediction value (≥71.0% sensitivity and ≥85.2% specificity) than existing tools—a knowledge-based tool, Derek (56.0% sensitivity and 86.9% specificity), and a statistics-based tool, MultiCase (91.0% sensitivity and 64.9% specificity) [7]. A direct comparison of prediction performances between previous and present studies is difficult because the training data set and prediction target were different, and other external databases do not have a misleading positives class. However, our study suggests that separation of misleading positive results from positive results may contribute to improvements found in both sensitivity and specificity.

Although structural alerts have been developed in several tools for both in vitro and in vivo chromosomal damages [44,45], these models could not separate positives and misleading positives. By contrast, our model enabled us to find partial structures (OFGs) within target chemicals, which are related to positives and misleading positives, and may distinguish misleading positives from previously identified positive chemicals. OFG is a simple piece of structural information and is very useful for predicting toxicity by toxicologists. Moreover, our virtual poly-clastogens visually indicated causal structures for misleading positives and positives using OFGs. In a study by Ashby et al. [31], poly-carcinogen and its concepts, which played crucial roles in toxicological evaluation, were reported using the emerging in silico tools. While not all causal factors have been considered, we believe that our concept of virtual poly-clastogens using OFGs will enable toxicologists to better understand features of chemical structures related to positives and misleading positives.

The suggested mechanisms of OFGs in in silico evaluation can aid in deciding test protocols and conditions to avoid the occurrence of misleading positives. For example, when testing chemicals containing OFGs related to low pH, it would be effective to add a buffer to the medium in advance [14]. Furthermore, we can select more realistic test conditions by considering the application method, e.g., a 3D skin model for cosmetic ingredients [46,47]. Thus, our model can both predict the results of new chemicals and be used to reevaluate analogs of past positives as positives or misleading positives. Because misleading positives of chemicals caused by excessive toxicity [12,41], metabolic overload [48], and oxidative stress [12,49] could induce positive results in in vitro test conditions [7,15], consideration of in vitro-specific conditions such as ADME and chemical properties (pH, molecular weight) could be effective to improve the predictivity of misleading positives in the future.

Although this model indicated a high level of accuracy, our model was applied only to internal cross-validation, and it has been calculated on the basis of an assumption that synthesized chemicals by SMOTE have similar properties. Since the accuracies of the model for the original data sets were equivalent to the synthesized chemicals, the model can predict chemical results accurately, at least within the range of current training data sets. However, additional modifications would be effective to improve the applicability of our model: (1) refining the OFG lists to decrease substances that cannot be analyzed and to grasp more specific structures for chromosomal damages; (2) adding data of tested chemicals to increase the density of the chemical space [9]; and (3) using a molecular descriptor to cover further chemical spaces and ADME [50]. Subsequently, external validation should be conducted, as previously recommended [25]. The review of data (e.g., Carcinogenicity Genotoxicity eXperience data set) and the recategorization of misleading positives by experts are needed, and this is one important step toward developing accurate in silico tools. In addition, combinatorial use with other in silico tools could further improve the applicability of our model [7].

To summarize, we developed a prediction model using OFGs and a virtual poly-clastogen and applied it to genotoxicity evaluation. Using the updated and reclassified training data, we achieved both higher sensitivity and specificity and were able to interpret mechanisms of action. The elimination of causal structures or substituting them with other nontoxic structures will allow us to develop new and safer chemicals without genotoxic concerns. Furthermore, our approach can contribute to future investigations of various toxicities resulting in different outcomes between in vitro and in vivo tests, enabling a quantitative structure–activity relationship to achieve precise in vitro–in vivo extrapolation. 

## Figures and Tables

**Table 1:** Prediction performances of the developed models.

	Accuracy (%)	Sensitivity (%)	Specificity (%)
P	85.6	72.6	92.7
N	80.3	71.0	85.2
MP	87.9	71.6	94.8

P: positives; N: negatives; MP: misleading positives. Average prediction rates are shown after 10-fold cross-validation for SMOTE data set.

**Table 2:** OFGs related to positive prediction.

OFG	Odds Ratio	Reported Main Toxicological Effect or Mechanisms Likely Related to Positive Results	REF
Epoxide	13.94	DNA binding (a)	[35]
Fused unsaturated carbocycles	10.84	metabolites: DNA binding (c) *	[21]
Alkoxysilane	10.21	DNA binding (a)	[11]
Sulfonate ester	9.16	DNA binding (a)	[35]
Fused heterocyclic aromatic	9.14	DNA intercalation (c)	[35]
N. Nitroso	9.09	DNA binding (a)	[35]
Amidine	8.34	DNA minor groove binders (b)	[36]
Isocyanate	8.34	DNA acylation (a)	[35]
Dianilines	8.28	DNA binding (c)	[21]

(**a**) This organic functional group (OFG) has been reported as a structural alert or causative factor. (**b**) Chemicals with a part of this OFG have been reported, although no direct information has been found on this OFG. (**c**) Structural alerts with a part of this OFG have been reported, although no direct information has been found on this OFG. * Metabolites were estimated for chemicals with this OFG using “in vivo rat metabolism simulator” in the OECD QSAR toolbox [21].

**Table 3:** OFGs related to misleading positive prediction.

OFG	Odds Ratio	Reported Main Toxicological Effects or Mechanisms Likely Related to Misleading Positive Results	REF
Oxazole/Izoxazole	12.32	Anti-tuberculosis activity (a)	[37]
Benzthiazolinone/Benzoisothiazolinone	11.83	Reaction with amino groups of lysine residues (b)	[38]
Phosphonium, salt	7.68	Cytotoxicity (b)	[39]
Acetoxy	4.09	Low pH (a)	[11]
Methacrylate	4.05	DNA reactivity in vitro-specific and/or cytotoxicity (b)	[11]

(**a**) Chemicals with a part of this OFG have been reported, although no direct information was found on this OFG. (**b**) This OFG has been reported as a structural alert or causative factor.

## Supplementary Materials

The following are available online at https://www.mdpi.com/2073-4425/11/10/1181/s1, Table S1: List of chemicals. Table S2: Feature selection by elastic net model. Figure S1: Graphical flow of in silico modeling in this study.
