# Supplementary material for: In Silico Model for Chemical-Induced Chromosomal Damages Elucidates Mode of Action and Irrelevant Positives
Source: Genes (Basel). 2020 Oct 11;11(10):1181. doi: 10.3390/genes11101181 (PMC7650694; doi:10.3390/genes11101181)
Supplement: Supplementary file 1 [file genes-11-01181-s001.zip › Table S1. List of chemicals.pdf]

Table S1. List of chemicals

Category	No.	CAS No.	Chemical name	
Positive result chemicals (positives)	1	50-32-8	Benzo[a]pyrene	*1
	2	53-96-3	2-Acetylaminofluorene	*1
	3	95-80-7	2,4-Diaminotoluene	*1
	4	1162-65-8	Aflatoxin B1	*1
	5	57-97-6	7,12-Dimethylbenzanthracene	*1
	6	123-31-9	Hydroquinone	*1
	7	66-27-3	MMS	*1
	8	62-75-9	Dimethylnitrosamine	*1
	9	759-73-9	ENU	*1
	10	106-47-8	p-Chloroaniline—free base and HCl salt	*1
	11	33419-42-0	Etoposide	*1
	12	76180-96-6	IQ (2-amino-3methylimidazo[4,5-f]quinoline)	*1
	13	6055-19-2	Cyclophosphamide	*1
	14	105650-23-5	PhIP.HCl (2-amino-1-methyl-6-phenylimidazo [4,5-b]pyridine	*1
	15	30516-87-1	Azidothymidine	*1
	16	33069-62-4	Taxol	*1
	17	50-07-7	Mitomycin C	*1
	18	56-57-5	4-Nitroquinoline-N-oxide	*1
	19	64-86-8	Colchicine	*1
	20	143-67-9	Vinblastine sulfate	*1
	21	147-94-4	Cytosine arabinoside	*1
	22	51-21-8	5-Fluorouracil	*1
	23	591-27-5	3-Aminophenol	*2
	24	123-30-8	4-Aminophenol	*2
	25	112-26-5	1,2-Bis(2-chloroethoxy)ethane	*2
	26	38640-62-9	Bis(1-methylethyl)naphthalene	*2
	27	95-31-8	N-tert-Butyl-2-benzothiazolesulfenamide	*2
	28	88-60-8	6-tert-Butyl-m-cresol	*2
	29	611-19-8	1-Chloro-2-(chloromethyl)benzene	*2
	30	1570-64-5	4-Chloro-o-cresol	*2
	31	87-84-3	Chloropentabromocyclohexane	*2
	32	106-48-9	4-Chlorophenol	*2
	33	91-76-9	2,4-Diamino-6-phenyl-s-triazine	*2
	34	109-64-8	1,3-Dibromopropane	*2
	35	760-23-6	3,4-Dichloro-1-butene	*2
	36	3209-22-1	1,2-Dichloro-3-nitrobenzene	*2
	37	89-61-2	1,4-Dichloro-2-nitrobenzene	*2
	38	4979-32-2	N,N-Dicyclohexyl-2-benzothiazolesulfenamide	*2
	39	298-06-6	O,O'-Diethyl dithiophosphate	*2
	40	623-91-6	Diethyl fumarate	*2
	41	2439-35-2	2-(Dimethylamino)ethyl acrylate	*2
	42	793-24-8	N-(1,3-Dimethylbutyl)-N'-phenyl-p-phenylenediamine	*2
	43	5124-25-4	Disperse Yellow 42	*2
	44	106-91-2	2,3-Epoxypropyl methacrylate	*2
	45	156-43-4	4-Ethoxybenzeneamine (p-phenetidin)	*2
	46	84-51-5	2-Ethylanthraquinone	*2
	47	123-07-9	4-Ethylphenol	*2
	48	126-98-7	Methacrylonitrile (methyl acrylonitrile)	*2
	49	4461-52-3	Methoxymethanol	*2
	50	101-14-4	4,4'-Methylenebis(2-chloroaniline)	*2
	51	1333-16-0	Methylenediphenol	*2
	52	556-61-6	Methyl isothiocyanate	*2
	53	2581-34-2	3-Methyl-4-nitrophenol	*2
	54	108-39-4	3-Methylphenol (m-cresol)	*2
	55	97-52-9	4-Nitro-o-anisidine	*2
	56	13936-21-5	2-Pentylanthraquinone	*2
	57	941-69-5	N-Phenylmaleimide	*2
	58	101-72-4	N-Phenyl-N'-isopropyl-p-phenylenediamine	*2
	59	118-79-6	2,4,6-Tribromophenol	*2
	60	100-69-6	2-Vinylpyridine	*2
	61	99-09-2	3-Nitrobenzenamine	*2
	62	111-41-1	N-(Aminoethyl)ethanolamine	*2
	63	81-16-3	2-Amino-1-naphthalenesulfonic acid	*2

	64	109-70-6	1-Bromo-3-chloropropane		*2
	65	2867-47-2	2-(Dimethylamino)ethyl methacrylate		*2
	66	87-59-2	2,3-Dimethylaniline		*2
	67	87-62-7	2,6-Dimethylaniline (2,6-xylidine)		*2
	68	108-69-0	3,5-Dimethylaniline (3,5-xylidine)		*2
	69	26630-87-5	Disperse Red 206		*2
	70	536-90-3	3-Methoxybenzeneamine		*2
	71	80-51-3	4,4'-Oxybis(benzenesulfonylhydrazide)		*2
	72	4189-44-0	Thiourea dioxide		*2
	73	26471-62-5	Tolylene diisocyanate (toluene diisocyanate)		*2
	74	88-89-1	2,4,6-Trinitrophenol (picric acid)		*2
	75	92-88-6	4,4'-Biphenyldiol		*2
	76	89-72-5	o-sec-Butylphenol		*2
	77	98-54-4	p-tert-Butylphenol		*2
	78	620-17-7	3-Ethylphenol		*2
	79	90-02-8	2-Hydroxybenzaldehyde		*2
	80	2216-69-5	1-Methoxynaphthalene		*2
	81	620-92-8	4,4'-Methylenediphenol		*2
	82	50957-96-5	Phosphoric acid, dodecyl ester, sodium salt		*2
	83	3048-65-5	3a,4,7,7a-Tetrahydro-1H-indene		*2
	84	108-73-6	1,3,5-Trihydroxybenezene		*2
	85	56-93-9	Benzyltrimethylammonium chloride		*2
	86	85-41-6	Phthalimide		*2
	87	88-18-6	2-tert-Butylphenol		*2, *3
	88	4286-23-1	4-(1-Methylethenyl)phenol		*2, *3
	89	26444-49-5	Diphenyl cresyl phosphate		*2
	90	95-50-1	o-Dichlorobenzene		*2, *3
	91	75-50-3	Trimethylamine		*2
	92	100-61-8	N-Methylaniline		*2
	93	78-97-7	2-Hydroxypropanenitrile		*2
	94	103-83-3	N,N-Dimethylbenzylamine		*2
	95	105-99-7	Dibutyl adipate		*2
	96	41267-43-0	C.I. Fluorescent brightner 271		*2
	97	2768-02-7	Ethenyltrimethoxysilane		*2
	98	27676-62-6	1,3,5-Tris(3,5-di-tertbutyl-4-hydroxybenzyl)isocyanuricacid		*2
	99	103-69-5	N-Ethylaniline		*2
	100	105-45-3	Methyl acetoacetate		*2
	101	121-45-9	Trimethoxyphosphine		*2
	102	583-39-1	2-Mercaptobenzimidazole		*2
	103	10108-64-2	Cadmium chloride	†	*1
	104	15663-27-1	Cisplatin	†	*1
	105	7784-46-5	Sodium arsenite	†	*1
	106	10022-68-1	Cadmium nitrate tetrahydrate	†	*2
	107	7789-12-0	Chromic acid disodium salt dihydrate	†	*2
	108	7803-57-8	Hydrazine monohydrate	†	*2
Negative result chemicals (negatives)	109	75-65-0	Tert-butyl alcohol		*1
	110	61-82-5	Amitrole		*1
	111	117-81-7	Di-(2-ethylhexyl)phthalate		*1
	112	67-72-1	Hexachloroethane		*1
	113	5989-27-5	D-Limonene		*1
	114	69-65-8	D-mannitol		*1
	115	108-78-1	Melamine		*1
	116	598-55-0	Methyl carbamate		*1
	117	999-81-5	(2-chloroethyl)trimethyl-ammoniumchloride		*1
	118	110-86-1	Pyridine		*1
	119	111-42-2	Diethanolamine		*1
	120	1212-29-9	N,N-dicyclohexyl thiourea		*1
	121	134-72-5	Ephedrinesulphate/hydrochloride		*1
	122	7177-48-2	Ampicillin trihydrate		*1
	123	643-22-1	Erythromycin/erythromycinstearate		*1
	124	834-28-6	Phenformin HCl		*1
	125	124937-51-5	Tolterodine		*1
	126	15307-79-6	Sodium diclofenac		*1
	127	68291-97-4	Zonisamide		*1
	128	91374-21-9	Ropinirole		*1

129	287714-41-4	Rosuvastatin	*1
130	97240-79-4	Topiramate	*1
131	107753-78-6	Zafirlukast	*1
132	82-45-1	1-Aminoanthraquinone	*2
133	115-70-8	2-Amino-2-ethyl-1,3-propanediol	*2
134	87-02-5	7-Amino-4-hydroxy-2-naphthalenesulfonic acid	*2
135	78-67-1	2,2'-Azobis(2-methylpropionitrile)	*2
136	1477-55-0	1,3-Bis(aminomethyl) benzene	*2
137	6731-36-8	1,1-Bis(tert-butyldioxy)-3,3,5-trimethylcyclohexane	*2
138	1552-42-7	3,3-Bis(p-dimethylaminophenyl)-6-dimethylaminophthalide	*2
139	103-24-2	Bis(2-ethylhexyl) azelate	*2
140	80-43-3	Bis(1-methyl-1-phenylethyl) peroxide	*2
141	110-30-5	1,2-Bis(staeroylamino) ethane	*2
142	584-03-2	1,2-Butanediol	*2
143	110-63-4	1,4-Butanediol	*2
144	7580-85-0	2-tert-Butoxyethanol	*2
145	88-85-7	2-sec-Butyl-4,6-dinitrophenol	*2
146	97-88-1	Butyl methacrylate	*2
147	1879-09-0	6-tert-Butyl-2,4-xylenol	*2
148	683-10-3	N-(Carboxymethy)-N,N-dimethyl-1-dodecanaminium, inner salt	*2
149	109-69-3	1-Chlorobutane	*2
150	6448-95-9	C.I.Pigment Red 22	*2
151	461-58-5	Cyanoguanidine	*2
152	100-54-9	3-Cyanopyridine	*2
153	110-83-8	Cyclohexene	*2
154	95-33-0	N-Cyclohexyl-2-benzothiazolesulfenamide	*2
155	5281-04-9	D&C Red No.7	*2
156	123-42-2	Diacetone alcohol	*2
157	526-78-3	2,3-Dibromosuccinic acid	*2
158	107-66-4	Dibutyl phosphate	*2
159	95-73-8	2,4-Dichloro-1-methylbenzene	*2
160	611-06-3	2,4-Dichloronitrobenzene	*2
161	118-69-4	2,6-Dichlorotoluene	*2
162	626-17-5	1,3-Dicyanobenzene	*2
163	623-26-7	1,4-Dicyanobenzene	*2
164	538-75-0	Dicyclohexylcarbodiimide	*2
165	77-73-6	Dicyclopentadiene	*2
166	211495-85-1	Dicyclopentylsilanediol	*2
167	105-05-5	1,4-Diethylbenzene	*2
168	28575-17-9	Diethylbiphenyl	*2
169	134-62-3	N,N-Diethyl-m-toluamide	*2
170	3648-21-3	Diheptyl phthalate	*2
171	111-03-5	2,3-Dihydroxypropyl 9-cis-octadecenoate	*2
172	25321-09-9	Diisopropylbenzene	*2
173	95-64-7	3,4-Dimethylaniline (3,4-Xylidine)	*2
174	840-65-3	Dimethyl 2,6-naphthalenedicarboxylate	*2
175	6165-51-1	1,4-Dimethyl-2-(1-phenylethyl) benzene	*2
176	126-30-7	2,2-Dimethyl-1,3-propandediol	*2
177	102-06-7	1,3-Diphenylguanidine	*2
178	882-33-7	Diphenyl disulfide	*2
179	1241-94-7	Diphenyl 2-ethylhexyl phosphate	*2
180	6106-21-4	Disodium succinate hexahydrate	*2
181	119-06-2	Ditridecyl phthalate	*2
182	1321-74-0	Divinylbenzene	*2
183	112-85-6	Docosanoic acid	*2
184	5707-44-8	4-Ethylbiphenyl	*2
185	688-84-6	2-Ethylhexyl methacrylate	*2
186	77-99-6	2-Ethyl-2-hydroxymeth-1,3-propanediol	*2
187	16219-75-3	5-Ethylidene-2-norbornene	*2
188	96-29-7	Ethyl methyl ketoxime	*2
189	100-74-3	4-Ethylmorpholine	*2
190	4390-04-9	2,2,4,4,6,8,8-Heptamethylnonane	*2
191	544-76-3	n-Hexadecane	*2
192	1843-05-6	2-Hydro-4-(octyloxy)benzophenone	*2
193	3846-71-7	2-(2'-Hydroxy-3',5'-di-tert-butylphenyl) benzotriazole	*2

194	96-45-7	2-Imidazolidinethione	*2
195	108-80-5	Isocyanuric acid	*2
196	79-94-7	4,4'-Isopropylidenebis (2,6-dibromophenol)	*2
197	79-39-0	Methacrylamide	*2
198	123-11-5	4-Methoxybenzaldehyde	*2
199	56539-66-3	3-Methoxy-3-methyl-1-butanol	*2
200	70-55-3	4-Methylbenzenesulfonamid	*2
201	111-82-0	Methyl dodecanoate	*2
202	98-83-9	1-Methylethenylbenzene	*2
203	99-88-7	4-(1-Methylethyl) aniline	*2
204	109-59-1	2-(1-Methylethoxy) ethanol	*2
205	121-03-9	2-Methyl-5-nitrobenzenesulfonic acid	*2
206	4457-71-0	3-Methyl-1,5-pentanediol	*2
207	691-37-2	4-Methy-1-pentene	*2
208	599-64-4	4-(1-Methyl-1-phenylethyl) phenol	*2
209	99-71-8	4-(1-Methylpropyl)phenol	*2
210	5460-09-3	Monosodium 4-amino-5-hydroxy-2,7-naphthalenedisulfonate	*2
211	6099-57-6	1-Naphthol-4-sulfonic acid sodium salt	*2
212	119-47-1	2,2'-Nethylenebis(6-tert-butyl-p-cresol)	*2
213	25154-52-3	Nonylphenol	*2
214	111-88-6	1-Octanethiol	*2
215	140-66-9	p-tert-Octylphenol	*2
216	629-62-9	n-Pentadecane	*2
217	115-77-5	Pentaerythritol	*2
218	7299-99-2	Pentaerythritol tetra(2-ethylhexanoate)	*2
219	3586-14-9	3-Phenoxytoluene	*2
220	147-14-8	Phthalocyanine Blue	*2
221	14832-14-5	Pigment Green No.7 (Hexadecachloro)	*2
222	1328-53-6	Pigment Green No.7 (Plychloro, unspecified)	*2
223	6505-28-8	Pigment Orange 16	*2
224	842-18-2	Potassium 7-hydroxy-1,3-naphthalenedisulfonate	*2
225	108-65-6	Propylene glycol monomethyl ether acetate	*2
226	12033-89-5	Silicone nitride	*2
227	130-13-2	Sodium 4-amino-1-naphthalenesuifonate	*2
228	4016-24-4	Sodium 1-methoxycarbonylpentadecane-2-sulfonate	*2
229	135-51-3	Sodium 2-naphthol-3,6-disulfonate	*2
230	127-68-4	Sodium 3-nitrobenzenesulfonate	*2
231	657-84-1	Sodium p-toluenesulfonate	*2
232	79-27-6	Tetrabromoethane	*2
233	97-99-4	Tetrahydrofurfuryl alcohol	*2
234	11070-44-3	Tetrahydromethyl-1,3-isobenzofuranedione	*2
235	126-33-0	Tetrahydrothiophene 1,1-dioxide	*2
236	75-59-2	Tetramethylammonium hydroxide	*2
237	96-69-5	4,4'-Thiobis(6-tert-butyl-m-cresol)	*2
238	111-17-1	3,3'-Thiobispropanoic acid	*2
239	110-02-1	Thiophene	*2
240	88-19-7	o-Toluenesulfonamide	*2
241	108-44-1	m-Toluidine	*2
242	98-08-8	Trifluoromethylbenzene	*2
243	526-73-8	1,2,3-Trimethylbenzene	*2
244	95-63-6	1,2,4-Trimethylbenzene	*2
245	6846-50-0	2,2,4-Trimethyl-1,3-pentanediol diisobutyrate	*2
246	512-56-1	Trimethyl phosphate	*2
247	1066-40-6	Trimethylsilanol	*2
248	89-04-3	Trioctylbenzene-1,2,4-tricarboxylate	*2
249	76-83-5	Triphenylchloromethane	*2
250	24800-44-0	Tripropylene glycol	*2
251	78-51-3	Tris(2-butoxyethyl) phosphate	*2
252	26967-76-0	Tris(p-cumenyl) phosphate	*2
253	3319-31-1	Tris(2-ethylhexyl) 1,2,4-benzenetricarboxylate	*2
254	78-42-2	Tris(2-ethylhexyl) phosphate	*2
255	839-90-7	1,3,5-Tris(2-hydroxyethyl)-1,3,5-triazine-2,4,6-(1H,3H,5H)-trione	*2
256	77-85-0	1,1,1-Tris(hydroxymethyl)ethane	*2
257	1025-15-6	1,3,5-Tris(2-propenyl) isocyanuric acid	*2
258	1120-21-4	Undecane	*2

	259	8007-18-9	C.I.Pigment Yellow 53	†	*2
	260	7550-35-8	Lithium bromide	†	*2
	261	39430-27-8	Nickel(II) carbonate hydroxide tetrahydrate	†	*2
	262	13472-30-5	Tetrasodium monosilicate hydrate	†	*2
	263	7756-94-7	Triisobutylene	†	*2
	264	122852-42-0	Alosetron	†	*1
	265	688046-61-9	Pyriofenone	†	*1
Misleading or irrelevant positive result chemicals (misleading positives)	266	27813-02-1	Methacrylic acid, monoester with propane-1,2-diol		*2
	267	106-37-6	1,4-Dibromobenzene		*2
	268	121-47-1	3-Aminobenzenesulfonic acid		*2
	269	88-53-9	2-Amino-5-chloro-4-methylbenzenesulfonic acid		*2
	270	88-44-8	2-Amino-5-methylbenzenesulfonic acid		*2
	271	102-76-1	Glycerol triacetate		*2
	272	99-96-7	4-Hydroxybenzoic acid		*2
	273	86-87-3	1-Naphthylacetic acid		*2
	274	585-07-9	tert-Butyl-methacrylate		*2
	275	101-83-7	Dicyclohexylamine		*2
	276	868-77-9	2-Hydroxyethyl methacrylate		*2
	277	91-15-6	1,2-Dicyanobenzene		*2
	278	105-16-8	2-(Diethylamino)ethyl methacrylate		*2
	279	5039-78-1	(Methacryloyloxyethyl)trimethylammonium chloride		*2
	280	95-57-8	2-Chlorophenol		*2
	281	102-81-8	2-(Di-n-butylamino)ethanol		*2
	282	51-28-5	2,4-Dinitrophenol		*2
	283	88-09-5	2-Ethylbutyric acid		*2
	284	824-78-2	p-Nitrophenol sodium salt		*2
	285	1338-41-6	Sorbitan monooctadecanoate		*2
	286	99-94-5	4-Methylbenzoic acid		*2, *3
	287	80-09-1	4,4'-Sulfonyldiphenol		*2, *3
	288	97-39-2	1,3-Bis(2-methylphenyl)guanidine		*2, *3
	289	2403-88-5	2,2,6,6-Tetramethyl-4-hydroxypiperidine		*2, *3
	290	83-32-9	Acenaphthene		*2, *3
	291	95-32-9	2-(4-Morpholinyldithio)benzothiazole		*2, *3
	292	2416-94-6	2,3,6-Trimethylphenol		*2, *3
	293	96-76-4	2,4-Di-tert-butylphenol		*2, *3
	294	93-68-5	o-Acetoacetotoluidine		*2, *3
	295	89-83-8	Thymol		*2, *3
	296	99-04-7	3-Methylzenzoic acid		*2, *3
	297	118-92-3	o-Anthranilic acid		*1
	298	100-51-6	Benzyl alcohol		*1
	299	536-33-4	Ethionamide		*1
	300	140-88-5	Ethyl acrylate		*1
	301	97-53-0	Eugenol		*1
	302	15356-70-4	d,l-Menthol		*1
	303	108-46-3	1,3-Dihydroxybenzene(resorcinol)		*1
	304	127-69-5	Sulfisoxazole		*1
	305	57-13-6	Urea		*1
	306	100-02-7	p-Nitrophenol		*1
	307	94-96-2	2-Ethyl-1,3-hexanediol		*1
	308	1948-33-0	Tertiarybutylhydroquinone		*1
	309	78-84-2	Isobutyraldehyde		*1
	310	128-44-9	Sodium saccharin		*1
	311	113-92-8	Chlorpheniramine maleate		*1
	312	124-64-1	Tetrakis(hydroxymethyl)phosphonium chloride		*1
	313	97-77-8	Tetraethylthiuramdisulfide [AKAdisulfiram]		*1
	314	13939-25-8	Triphosphoric acid aluminium salt	†	*2
	315	7782-63-0	Ferrous sulfate heptahydrate	†	*2

*1 Kirkland 2016; *2 Morita 2012; *3 Fujita 2016

† These chemicals were eliminated for model development because they did not have OFGs.
