# Supplementary figures and images for: In Silico Model for Chemical-Induced Chromosomal Damages Elucidates Mode of Action and Irrelevant Positives
Source: Genes (Basel). 2020 Oct 11;11(10):1181. doi: 10.3390/genes11101181 (PMC7650694; doi:10.3390/genes11101181)

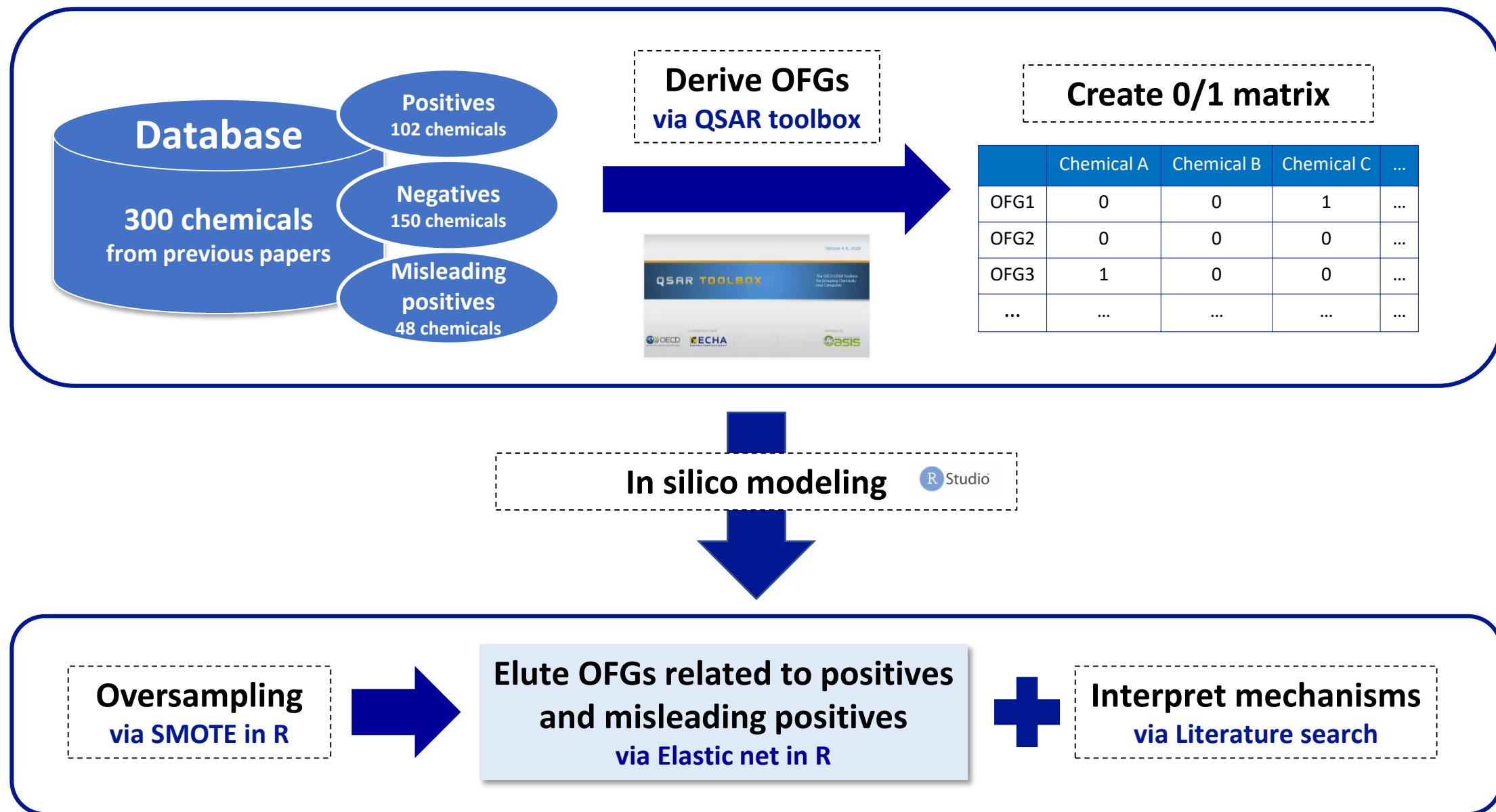

Figure S1. Graphical flow of *in silico* modeling in this study.

Supplement: Supplementary file 1 [file genes-11-01181-s001.zip › Figure S1. Graphical flow of in silico modeling in this study.pdf]
